# Atomistic Compositional Details and Their Importance for Spin Qubits in Isotope‐Purified Silicon Quantum Wells

**DOI:** 10.1002/advs.202407442

**Published:** 2024-09-11

**Authors:** Jan Klos, Jan Tröger, Jens Keutgen, Merritt P. Losert, Nikolay V. Abrosimov, Joachim Knoch, Hartmut Bracht, Susan N. Coppersmith, Mark Friesen, Oana Cojocaru‐Mirédin, Lars R. Schreiber, Dominique Bougeard

**Affiliations:** ^1^ JARA‐FIT Institute for Quantum Information Forschungszentrum Jülich GmbH & RWTH Aachen University 52074 Aachen Germany; ^2^ Institute of Materials Physics University of Münster 48149 Münster Germany; ^3^ Tascon GmbH 48149 Münster Germany; ^4^ I. Physikalisches Institut IA RWTH Aachen University 52074 Aachen Germany; ^5^ University of Wisconsin‐Madison Madison WI 53706 USA; ^6^ Leibniz‐Institut für Kristallzüchtung (IKZ) 12485 Berlin Germany; ^7^ Institute of Semiconductor Electronics RWTH Aachen University 52074 Aachen Germany; ^8^ University of New South Wales Sydney 2025 Australia; ^9^ INATECH, Albert‐Ludwigs Universität Freiburg 79110 Freiburg im Breisgau Germany; ^10^ ARQUE Systems GmbH 52074 Aachen Germany; ^11^ Institut für Experimentelle und Angewandte Physik Universität Regensburg 93040 Regensburg Germany

**Keywords:** spin qubit, valley splitting, atom probe tomography, silicon‐germanium heterostructure, isotope‐purified silicon, molecular beam epitaxy, segregation, diffusion, quantum computing

## Abstract

Understanding crystal characteristics down to the atomistic level increasingly emerges as a crucial insight for creating solid state platforms for qubits with reproducible and homogeneous properties. Here, isotope concentration depth profiles in a SiGe/^28^Si/SiGe heterostructure are analyzed with atom probe tomography (APT) and time‐of‐flight secondary‐ion mass spectrometry down to their respective limits of isotope concentrations and depth resolution. Spin‐echo dephasing times $T_{2}^{\mathrm{echo}}=128\;\mu s$ and valley energy splittings *E*_VS_ around 200μeV have been observed for single spin qubits in this quantum well (QW) heterostructure, pointing toward the suppression of qubit decoherence through hyperfine interaction with crystal host nuclear spins or via scattering between valley states. The concentration of nuclear spin‐carrying ^29^Si is 50 ± 20ppm in the ^28^Si QW. The resolution limits of APT allow to uncover that both the SiGe/^28^Si and the ^28^Si/SiGe interfaces of the QW are shaped by epitaxial growth front segregation signatures on a few monolayer scale. A subsequent thermal treatment, representative of the thermal budget experienced by the heterostructure during qubit device processing, broadens the top SiGe/^28^Si QW interface by about two monolayers, while the width of the bottom ^28^Si/SiGe interface remains unchanged. Using a tight‐binding model including SiGe alloy disorder, these experimental results suggest that the combination of the slightly thermally broadened top interface and of a minimal Ge concentration of 0.3% in the QW, resulting from segregation, is instrumental for the observed large $E_{\mathrm{VS}}=200\;\mu eV$. Minimal Ge additions <1%, which get more likely in thin QWs, will hence support high *E*_VS_ without compromising coherence times. At the same time, taking thermal treatments during device processing as well as the occurrence of crystal growth characteristics into account seems important for the design of reproducible qubit properties.

## Graphical abstract

Understanding crystal characteristics increasingly emerges as a crucial insight for creating solid state platforms for qubits with reproducible and homogeneous properties. Here, isotope concentration depth profiles in a SiGe/^28^Si/SiGe heterostructure are analyzed down to the atomistic level. Reasons for the observation of large valley splitting energies in this heterostructure are unveiled and implications of the observed concentration profiles for spin qubits discussed.

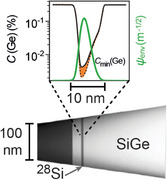

## 1 Introduction

The development of novel quantum technologies in condensed matter ‐ and in particular the quest for a scalable and fault‐tolerant quantum computer (QC) – increasingly ties decisive device performance parameters, such as the qubit fidelities and coherence times, to atomistic scale material properties. This strongly increases the need to get quantitative insights and, ideally, to find ways to control and custom‐tailor atomistic details of condensed matter. Lately, obtaining chemical and spatial information at interfaces with atomistic precision has been pointed out to play a paramount role in the development of large‐scale quantum information processors based on all major envisaged solid‐state qubits such as superconducting qubits, spin qubits in quantum dots (QDs) or color centers, and topological qubits.^[^ ^1^, ^2^, ^3^, ^4^ ^]^

Thin‐film epitaxially‐grown ^28^Si/SiGe heterostructures consisting of a tensile strained, isotope‐purified ^28^Si quantum well (QW) layer, sandwiched between two layers of the alloy SiGe, have been proven to be an excellent host for spin qubits.^[^ ^5^, ^6^ ^]^ These qubits are realized by controlling single to few electron spins in electrostatically‐defined quantum dots in the QW.^[^ ^7^, ^8^ ^]^ Important ingredients for a fault‐tolerant QC are long spin decoherence times, single and two‐qubit gates as well as fast spin detection; all with fidelities beyond the quantum error correction threshold.^[^ ^9^, ^10^, ^11^, ^12^, ^13^, ^14^ ^]^ The need of medium‐distance quantum information transfer^[^ ^15^ ^]^ triggered research on coherent transport of spin qubits using few operation signals,^[^ ^16^, ^17^, ^18^ ^]^ which poses high demand on material homogeneity.^[^ ^19^ ^]^ All these studies suggest that materials properties need to be understood on an atomistic scale to enable the realization of a ^28^Si/SiGe‐based large‐scale solid‐state QC.^[^ ^20^ ^]^ In particular, the atomistic details of the semiconductor heterostructure seem to be highly relevant for two sources of qubit decoherence: hyperfine interaction of the free electron spin qubit with nuclear spins of the host crystal lattice and intervalley spin decoherence in the ^28^Si QW. The relevant hyperfine contact interaction depends on the concentration of lattice atoms carrying non‐zero nuclear spins in the ^28^Si QW, with which the electron wavefunction of a considered spin qubit overlaps.^[^ ^21^ ^]^ This concentration is influenced by the degree of isotope purification in the ^28^Si QW, possibly by the ^28^Si/SiGe heterostructure epitaxy or also post‐growth bulk diffusion processes, thermally triggered for example during the qubit device fabrication.^[^ ^22^ ^]^ The intervalley spin decoherence, on the other hand, is particularly relevant in the case of a non‐desirable, uncontrolled occupation of an excited valley state during a spin qubit operation in the valley ground state.^[^ ^19^, ^23^, ^24^ ^]^ The relevant metric is the valley‐splitting energy (*E*_VS_) between the excited and the ground valley state, which needs to be sufficiently large for low‐decoherence qubit operation. The atomistic details of ^28^Si/SiGe interfaces such as atomic steps and SiGe alloy disorder^[^ ^25^, ^26^, ^27^, ^28^, ^29^, ^30^, ^31^, ^32^, ^33^, ^34^, ^35^, ^36^, ^37^, ^38^ ^]^ have been pointed out to be highly relevant for the magnitude of *E*_VS_ in a heterostructure, correlating with a significant spreading of experimentally determined valley energy splittings reported in the literature in various heterostructures.^[^ ^23^, ^30^, ^33^, ^39^, ^40^, ^41^, ^42^, ^43^, ^44^, ^45^, ^46^, ^47^, ^48^, ^49^, ^50^ ^]^ Being able to analyze spatial depth concentration profiles down to the few atomistic monolayers and the few 100 ppm concentration level of isotopes in as‐grown heterostructures as well as in processed devices is hence of importance to devise and test strategies to produce devices with well‐controlled *E*_VS_.

Here, we analyze a Si_0.7_Ge_0.3_/^28^Si/Si_0.7_Ge_0.3_ heterostructure grown by solid‐source molecular beam epitaxy (MBE, see Experimental Section). Our focus lies on the depth‐resolution of the composition profiles at the interfaces between ^28^Si and Si_0.7_Ge_0.3_ and on the isotope concentration of ^28^Si investigated with pulsed laser atom probe tomography (APT) and time‐of‐flight secondary‐ion mass spectrometry (ToF‐SIMS). We compare as‐grown samples with post‐growth annealed samples. The post‐growth annealing is representative of the highest thermal budget used during qubit device processing (see Experimental Section). Spin qubit devices processed from the same Si_0.7_Ge_0.3_/^28^Si/Si_0.7_Ge_0.3_ heterostructure used in the study presented here have previously been shown to feature excellent properties in terms of single spin qubit robustness, with a valley splitting energy *E*_VS_ ranging from 185μeV to 212μeV,^[^ ^46^ ^]^ an ensemble spin dephasing time $T_{2}^{*}\approx 20\;\mu s$ and a spin‐echo dephasing time $T_{2}^{\mathrm{echo}}=128\;\mu s$. Both dephasing times were not limited by the hyperfine contact interaction of residual ^29^Si isotopes in the QW.^[^ ^51^ ^]^

We find <60ppm nuclear spin‐carrying ^29^Si in the ^28^Si QW by APT, confirming the absence of isotope diffusion during the epitaxy or post‐growth anneal and in line with the qubit coherence times observed in devices made from this heterostructure. APT allows us to resolve a slight broadening of the top Si_0.7_Ge_0.3_/^28^Si QW interface by approximately two monolayers (1 ML = 0.132 nm) after a thermal anneal representative of device processing, compared to the as‐grown interface. Furthermore, our analysis uncovers slight signatures of segregation that occurred at the crystal growth front during the epitaxy of both interfaces, the Si_0.7_Ge_0.3_/^28^Si top and the ^28^Si/Si_0.7_Ge_0.3_ bottom interfaces. Moreover, our APT analysis suggests that the segregation at the bottom interface may result in the lowest Ge concentration reached in the 10.5 nm thick ^28^Si QW to be at most 0.3%. Using a tight‐binding and an effective‐mass model, we find that slight details of the experimental Ge concentration profiles, such as the comparatively subtle effects of the post‐growth annealing, seem to induce valley splitting energies around 200μeV.

## 2 Concentration Profile of the SiGe/^28^Si/SiGe Quantum Well

### 2.1 Heterostructure Characteristics and Measurement Conditions

**Figure 1:**
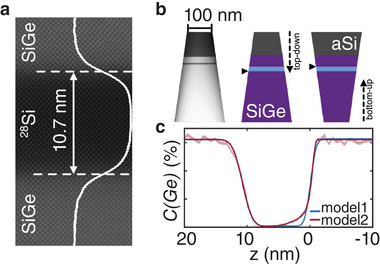
a) High resolution HAADF STEM image of the ^28^Si/SiGe heterostructure with an intensity profile obtained on all detected atomic columns. b) APT tip and two schematic tip configurations used to probe the top interface (bottom–up) and bottom interface (top–down) of the QW. c) Ge concentration profile obtained by APT across the QW, fitted by Equation (2) (model1) and by Equation (2) and Equation (3) (model2) respectively (see Experimental Section).

The Si_0.7_Ge_0.3_/^28^Si/Si_0.7_Ge_0.3_ QW part of the heterostructure has been grown at a nominal temperature of 350 °C with SiGe potential barriers of natural isotope composition and a QW highly purified in ^28^Si (see Experimental Section). **Figure** **1**a shows a high‐angle annular dark field scanning transmission electron micrograph (HAADF STEM) of the QW. The laterally averaged profile intensity (shown as an overlay in Figure 1a) and the performed ToF‐SIMS analysis (see Supporting Information) agree in a measured QW thickness of 10.5 ± 0.2 nm. This value of the QW thickness was used in APT data analysis, in addition to the correction method by Vurpillot et al.^[^ ^52^ ^]^ and the Landmark reconstruction,^[^ ^53^ ^]^ to obtain a precise 3D reconstruction of the heterostructure (see Experimental Section). For the Landmark reconstruction, a 15 atom‐percent Ge isosurface is used. Moreover, it has been shown^[^ ^54^, ^55^, ^56^ ^]^ that collecting an APT profile from SiGe across an interface with Si is less precise than collecting it from Si across the interface with SiGe, because of the different evaporation fields of each material. Hence, to ensure highest resolution, we have produced dedicated specimen tips (Figure 1b) for the APT analysis of each QW interface, to always probe a heterostructure interface from Si across SiGe. Thus, the bottom interface is analyzed using a specimen tip probed in a top‐down, while the top interface is analyzed using a specimen tip probed in a bottom‐up configuration, as sketched by arrows in Figure 1b and conducted in Ref. [55]. We measured two tips of each type and compared the results with another set of, in total, four specimens, which we annealed prior to preparing the tips (see Experimental Section). The interface broadenings of the Si and Ge profiles at the top and bottom QW interfaces determined from each individual analyzed tip are provided in the Supporting Information.

To determine interface profile widths, we fit the experimental, depth‐resolved concentration profiles with an error‐function model which has been widely used for the analysis of isotope profiles.^[^ ^57^, ^58^, ^59^ ^]^ For all TEM, ToF‐SIMS and APT experimental profiles, we chose to fit the top and bottom interface together within one profile by using a model with two opposing error functions, to suppress numerical errors (see Experimental Section). As discussed below, for APT profiles, we extend the error function fit at the bottom interface to better take into account a self‐limiting effect of Ge segregation during growth of a Si/SiGe interface.^[^ ^60^, ^61^ ^]^ Both fit models are shown on an exemplary APT profile in Figure 1c and are discussed in the Experimental Section.

In the following, *C*(*X*) denotes the depth‐resolved composition profile of *X*, while *C*(*z*; *X*) is the composition value of *X* at the depth *z* in growth direction. Along the manuscript, *X* will either be *Si* (standing here for the sum of the composition in the isotopes ^29, 30^Si), *Ge* (standing for the sum of the composition in the isotopes ^70, 72, 73, 74, 76^Ge) or a specific isotope, in particular ^28^ *Si*. Also, $r_{t;b}^{\prime }\left(X\right)$ stands for the interface width of the profile of *X* at a QW interface in the as‐grown heterostructure, while *r*_t; b_(*X*) represents the post‐growth annealed counterpart, with *t* for the top or *b* for the bottom QW interface.

### 2.2 Experimental Analysis of the SiGe/^28^Si Top Interface

**Figure 2:**
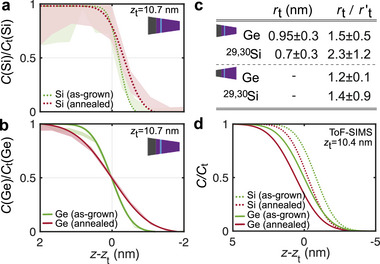
APT and ToF‐SIMS measurements of the top SiGe/^28^Si interface. Shaded areas in the profiles correspond to a single standard deviation interval of the measured data. a) APT data and fit of the two isotopes ^29, 30^Si concentration profile around the position of the top‐interface *z*_t_ and normalized to the fitted Si concentration $C_{t}^{\mathrm{Si}}$ of the top‐barrier. The inset sketches the bottom‐up APT specimen tips used for a precise analysis of the top SiGe/^28^Si interface (cf. Figure 1c). b) APT data as in panel **a**, here for the concentration profile of Ge. c) Table of the APT interface widths *r*_t_ for the annealed samples and of their ratio with the as‐grown width $r_{t}^{\prime }$. Upper half: Fit parameters resulting from data obtained in bottom‐up analysis, which is more precise for this top QW interface. Lower half: Comparison to ratio values obtained in top–down analysis, which is less precise for this top QW interface. d) Corresponding concentration profiles measured by ToF‐SIMS.

**Figure** **2** displays the depth‐resolved composition profiles across the SiGe/^28^Si top interface by APT and ToF‐SIMS and the corresponding parts of the fits to the profiles. For highest resolution of this top interface, we plot results of the APT specimens analyzed in bottom‐up configuration.

We first consider the depth‐resolved concentration profile *C*(Si) along the growth direction: Figure 2a shows this profile for the as‐grown and for the post‐growth annealed samples respectively. The profile of the as‐grown sample is well fitted by our error‐function model (see Equation 2 in the Experimental Section). The fit yields an interface width $r_{t}^{\prime }\left(\mathrm{Si}\right)=0.31\pm 0.09\;nm$ which corresponds to 2.4 monolayers of ^28^Si tensile strained by relaxed Si_0.7_Ge_0.3_. We postulate that $r_{t}^{\prime }\left(\mathrm{Si}\right)=0.31\pm 0.09\;nm$ is approaching the resolution limit of our APT measurement and of the 3D reconstruction, because an efficient mechanism which may cause a significant broadening of the top Si composition interface profile is not evident: The presence of segregation at the growth front during epitaxy will only concern subsurface Ge atoms, which may exchange with surface Si atoms, but there is no driving force for up‐floating of Si atoms in the growth direction.^[^ ^61^ ^]^ Also, there is no driving force for spontaneous intermixing of Si isotopes across a ^28^Si/^*nat*^SiGe interface.^[^ ^62^ ^]^ Furthermore no significant thermally‐driven diffusion is to be expected at the growth temperature of 350 °C.^[^ ^57^, ^58^, ^62^ ^]^

Figure 2b depicts the Ge profile *C*(Ge) around the top QW interface in the as‐grown and in the annealed samples. With $r_{t}^{\prime }\left(\mathrm{Ge}\right)=0.65\pm 0.07\;nm$ it is slightly larger than the interface width $r_{t}^{\prime }\left(Si\right)$ discussed in Figure 2a. We attribute this interface broadening to the segregation of Ge atoms, expected for a SiGe growth front overgrowing Si (here ^28^Si) in ultra high vacuum epitaxy, termed as leading edge in the literature. ^[^ ^60^, ^61^, ^63^, ^64^, ^65^, ^66^ ^]^ Considering $r_{t}^{\prime }\left(\mathrm{Si}\right)=0.31\pm 0.09\;nm$ to approach the resolution limit of our APT profiles, as discussed above, this broadening of the interface due to Ge segregation may be as low as $r_{t}^{\prime }\left(\mathrm{Ge}\right)-r_{t}^{\prime }\left(\mathrm{Si}\right)=0.34\pm 0.16\;nm$, which corresponds to 2.3 monolayers of relaxed Si_0.7_Ge_0.3_. The interface width of the APT Ge profile is comparable to recent APT studies of as‐grown structures produced in chemical vapor deposition (CVD)^[^ ^33^, ^55^, ^56^ ^]^ (see the Experimental Section for a conversion between interface width parameterizations). Note that segregation may be hampered under certain CVD growth conditions and that segregation is not discussed in these latter works.

In addition to the slight broadening of the Ge profile, we observe a second evidence of Ge leading edge segregation at the as‐grown SiGe/^28^Si top interface of the QW: the turning point of the Ge profile ($C_{\mathrm{Ge}}/C_{t}^{\mathrm{Ge}}=0.5$) is slightly shifted compared to the Si profile, as a consequence of the tendency of Ge atoms to float upon the growth front, up to a certain equilibrium concentration.^[^ ^61^ ^]^ Comparing Figure 2a,b, the average shift is 0.16 ± 0.11 nm for the APT profiles of as‐grown samples. To our knowledge, the experimental observation of this shifted turning point between Ge and Si in the presence of leading edge segregation has not been reported before.

For comparison to the as‐grown Si and Ge profiles, Figure 2a,b also show the profiles of post‐growth annealed samples, which represent a realistic thermal budget during qubit device processing (see Experimental Section). The Ge and Si profiles clearly reveal a broadening at the interface which we attribute to an isotropic bulk diffusion of Ge and Si in the heterostructure crystal during post‐growth annealing. Since diffusion of Ge and Si are negligible at 700 °C for 15 s in a SiGe and a Si crystal lattice, both considered at their thermodynamic equilibrium,^[^ ^57^, ^58^ ^]^ we attribute the observation of this annealing effect to the fact that epitaxial thin film growth from the gas phase (such as MBE or CVD) generally produces crystal lattices that do not perfectly match the thermodynamic crystal lattice equilibrium. For our heterostructures, it seems plausible that for example the QW growth temperature of 350 °C (see Experimental Section) and the strain in the Si QW favor an increased incorporation of vacancies or interstitial atoms into the as‐grown film compared to a lattice at the thermodynamic equilibrium. Under post growth annealing, non‐equilibrium vacancies or interstitial atoms will promote diffusion, to make the lattice tend toward its thermodynamic equilibrium. A signature of this equilibration is the slight profile widening which we experimentally resolve via APT in the Ge and the Si profiles. The average interface width of the Ge profile is *r*_t_(Ge) = 0.95 ± 0.3 nm, showing a larger variation between the results of the two specimens (*r*_t_(Ge) = [0.76 ± 0.03 nm, 1.13 ± 0.05 nm], see Supporting Information) than for the as‐grown specimen. The interfacial broadening of the Si profile is *r*_t_(Si) = 0.7 ± 0.3 nm, again with some variation among the two specimens (*r*_t_ = [0.5 ± 0.1 nm, 0.8 ± 0.1 nm], see Supporting Information). If we consider $r_{t}^{\prime }\left(\mathrm{Si}\right)=0.31\pm 0.09\;nm$ to approach the resolution limit of our APT analysis, as discussed earlier, $r_{t}\left(\mathrm{Ge}\right)-r_{t}^{\prime }\left(\mathrm{Si}\right)=0.7\pm 0.5\;nm$ represents the broadening of the Ge profile due to post‐growth annealing. The table in Figure 2c summarizes the interface widths *r*_t_ after the post‐growth anneal and the ratio $r_{t}/r_{t}^{\prime }$ between post‐growth annealed and as‐grown samples. As it is expected for the bulk diffusion in the heterostructure crystal, we find similar values for the ratios $r_{t}/r_{t}^{\prime }$ for Ge and Si. In the lower half of the table in in Figure 2c, we also mention the ratios $r_{t}/r_{t}^{\prime }$ determined on APT needles analyzed in the top‐down configuration. Although these conditions are less precise for the analysis of the top QW interface, the ratios for Ge and Si confirm the broadening of the top QW interfaces.

Figure 2d shows the Ge and Si profiles acquired by ToF‐SIMS, both for as‐grown and post‐growth annealed samples. The fitted interface widths *r*_t_ and $r_{t}^{\prime }$ for Ge and Si with values higher than 1.5 nm significantly exceed the interface widths obtained from APT. No significant broadening of the interface by annealing is evident in ToF‐SIMS, demonstrating a lower depth resolution compared to APT. However, ToF‐SIMS offers a better signal‐to‐noise ratio for individual data points of the concentration profile than APT due to the larger probe area of 100μm×100μm, which we integrate to calculate *C*(*z*, *Ge*) and *C*(*z*, *Si*). Notably, the probe area of APT corresponds to the size of a quantum dot^[^ ^46^, ^51^ ^]^ and is thus representative for the environment to which a single spin qubit is exposed. In the ToF‐SIMS crater, a RMS roughness of 3.2 nm is detected by atom force microscopy (see Supporting Information). This roughness dominates the interface widths in the measured concentration profiles. We attribute a significant part of this roughness in the crater to the presence of cross‐hatching in this type of heterostructure as discussed in the Supporting Information. The cross‐hatching, which manifests as a regular terracing on a μm scale in two perpendicular crystal directions, results from the strain relaxation via dislocations in the concentration‐graded buffer part of the heterostructure and is, hence, unavoidable. It thus seems that we have reached the resolution limit of ToF‐SIMS in terms of the quantification of the interface width in this type of heterostructure, presumably due to cross‐hatching in our layer structure. Note that also the HAADF‐STEM analysis results in a broader interface width compared to APT (Figure 1).

Summarizing the comparison between as‐grown and post‐growth annealed samples, we observe a post‐growth diffusional broadening of the Ge and Si profiles caused by annealing. The slight profile broadening is resolved by means of APT but not by ToF‐SIMS measurements. Both APT and ToF‐SIMS reveal signatures of segregation of Ge during the growth process, which becomes evident in a slightly retarded onset of the Ge compared to the Si profile for the top Si_0.7_Ge_0.3_/^28^Si interface.

### 2.3 Experimental Analysis of the ^28^Si/SiGe Bottom Interface

**Figure 3:**
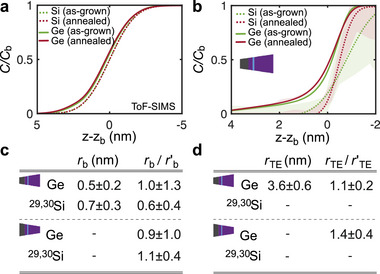
APT and ToF‐SIMS analysis of the bottom interface. Shaded areas in the profiles correspond to a single standard deviation interval of the measured data. a) ToF‐SIMS data for Si and Ge of the bottom QW interface for as‐grown and annealed samples. b) APT data for Si and Ge of the bottom QW interface for as‐grown and annealed samples. The inset sketches the analysis conditions in the top–down configuration, more precise for the bottom QW interface. c) Table of the APT interface widths *r*_b_ for the annealed samples and of their ratio with the as‐grown width $r_{b}^{\prime }$. Upper half: Fit parameters resulting from data obtained in top‐down analysis, which is more precise for this bottom QW interface. Lower half: Comparison to ratio values obtained in bottom‐up analysis, which is less precise for this bottom QW interface. d) Same as in (c) for the Ge trailing edge width *r*_TE_ (see Equation 3).

**Figure** **3** displays the depth‐resolved composition profiles across the ^28^Si/Si_0.7_Ge_0.3_ bottom QW interface by ToF‐SIMS and APT as well as the corresponding fits to the profiles. For highest resolution of this interface, we we plot results of the APT specimens analyzed in top‐down configuration.

Figure 3a shows the Ge‐ and the Si profiles acquired by ToF‐SIMS, both for as‐grown and post‐growth annealed samples. We find these profiles to be very similar to those observed for the top interface in Figure 2d. Indeed, all profiles are accurately described with a simple error‐function, following the model given by Equation (2) in the Experimental Section. Note, however, that for a Si overgrowth of Ge or SiGe, a segregation of Ge atoms into the Si overgrowth layer has been reported and experimentally resolved for as‐grown MBE structures grown at higher substrate temperatures than our heterostructure in the past.^[^ ^60^, ^61^, ^64^ ^]^ During Si overgrowth of SiGe, a self‐limiting mechanism of Ge segregation at the growth front is invoked,^[^ ^61^ ^]^ leading to a stretched Ge concentration profile, termed as trailing edge in the literature.^[^ ^60^ ^]^ We conclude that our experiments show that a Ge segregation and in particular its self‐limiting character for ^28^Si overgrowth of SiGe at the bottom interface cannot be resolved via ToF‐SIMS for this QW grown at 350 °C.

In contrast, a stretched Ge profile at the QW bottom interface is clearly resolved by means of APT as shown in Figure 3b. While the Si profiles *C*(Si) are accurately described with the error‐function of Equation 2 (see Experimental Section), this is not the case for the Ge profiles *C*(Ge). After a drop in Ge concentration, from a certain threshold value, the decrease in the Ge concentration is less pronounced. This is indicative of a self‐limitation of Ge segregation with increasing Si neighboring and corresponds to the trailing edge discussed in the literature. To our knowledge, this is the most narrow trailing edge (as a consequence of the comparatively low substrate temperature in the epitaxy) that has been experimentally resolved. The comparison between ToF‐SIMS and APT shows that such narrow trailing edges are only resolved in APT in these strain‐relaxed heterostructures. To accurately describe the trailing edge, we extend our error function model, as discussed in Equation (3) in the Experimental Section. In addition to the interface width $r_{b}^{\prime }\left(\mathrm{Ge}\right)$, we introduce a characteristic length $r_{\mathrm{TE}}^{\prime }\left(\mathrm{Ge}\right)$, to quantify the self‐limited segregation in the region of the trailing edge. For the as‐grown structures our Ge trailing edge profiles are in line with model predictions for segregation in ultra high vacuum epitaxy at substrate temperatures of 350 °C.^[^ ^61^ ^]^ Note that from the fit of the experimental APT Ge concentration profile as parameterized by Equations (2) and (3), we find a non‐zero minimum Ge concentration *C*_*min*_(Ge) in the QW: From *C*_0_(Ge) we deduce $C_{min}\left(\mathrm{Ge}\right)=0.21$% in the as‐grown heterostructure, which we interpret as an additional manifestation, here in our fit model, of the presence of the segregation trailing edge.

To analyze the effect of post‐growth annealing, we summarize the values of *r*_b_ and the ratios $r_{b}/r_{b}^{\prime }$ for Ge and Si in the table of Figure 3c as well as *r*_TE_ and $r_{\mathrm{TE}}/r_{\mathrm{TE}}^{\prime }$ for Ge in the table of Figure 3d. We also include the ratios determined from the less precise^[^ ^55^ ^]^ APT specimen configuration (here bottom‐up tips) to increase the statistics in the determination of the ratios. In contrast to the clear trend observed in the APT profiles of the Ge and Si at the top interfaces, the interfacial broadening of Si and Ge at the bottom interface is not significantly affected by post‐growth annealing, as seen for Ge and Si from the overlapping standard deviations of the as‐grown and of the annealed data in the whole range of analysis in Figure 3b (shaded areas) and from the tables in Figure 3c. Note that, as a consequence, the difference in interface width suggested by the Si profiles in Figure 3b is not physically meaningful, given the error bar. The comparatively large error bar is a consequence of the fact that we have to work with ^29^Si and ^30^Si to analyze the Si profiles in the heterostructure. These two isotopes represent only 7.8% of the Si atoms of the SiGe barrier. Regarding the additional fit parameter *C*_0_(Ge) (Equation 2), we deduce a value of $C_{min}\left(\mathrm{Ge}\right)=0.42$% in the annealed heterostructure, compared to $C_{min}\left(\mathrm{Ge}\right)=0.21$% in the as‐grown structure, indicative of very slight bulk diffusion during the post growth anneal. Note that the ToF‐SIMS analysis also does not display any difference between the as‐grown and the post‐growth annealed samples, as is expected, given the roughness‐limited depth resolution addressed in the analysis of the top interface.

### 2.4 Quantification of the Si Isotope and of the Minimal Ge Concentration in the QW

Going beyond the capabilities of APT in terms of depth resolution, we also explore the limits of the method in terms of the minimal resolvable Si isotope composition and minimal Ge composition in the ^28^Si QW. To ensure a high accuracy in the determination of the isotope composition, we increased the sensitivity to detect ^29^Si and ^30^Si ions inside the ^28^Si QW by employing a horizontal APT needle configuration (see Experimental Section) instead of the bottom–up or top–down geometries used before. We also describe the calibration of our measurement in the Experimental Section, using a piece of the ^28^Si MBE crystal source as a reference and the ^14.5^Si/^14^Si ratio of the double charged isotopes to avoid an interference with the mass‐to‐charge state 29 induced by ^28^SiH.

We find a composition of 50 ± 20ppm of ^29^Si in the QW by APT. This is in excellent agreement with the 41 ppm composition we determined for the ^28^Si MBE source crystal and suggests that no modification or contamination of the Si isotope enrichment takes place during the evaporation of the source crystal in MBE.^[^ ^57^, ^58^ ^]^ The composition of the ^30^Si isotope drops to 10ppm inside the ^28^Si layer. In the source crystal it was previously determined to be 1.3ppm(= 1.3 × 10^−4^%), in a different measurement.^[^ ^67^ ^]^ We conclude that 10ppm represents our detection limit of APT for Si isotopes in the QW layer. This proves the high detection capability of APT even in very confined space such as a 10.5 nm thick QW layer.

The non‐annealed APT needle in horizontal configuration is also advantageous to determine the minimal detectable Ge concentration in the QW. We determine a Ge concentration of 0.3%, which we consider as the upper boundary of the Ge concentration within the 10.5 nm QW, considering the detection limit of APT for the horizontal needle configuration. This experimental value is in good agreement with the fit results for the minimum Ge concentration in the QW *C*_*min*_(Ge) deduced from *C*_0_(Ge) in Equation (2) for the as‐grown ($C_{min}\left(\mathrm{Ge}\right)=0.21$%) and the annealed ($C_{min}\left(\mathrm{Ge}\right)=0.42$%) samples.

## 3 Valley Splitting Estimates for the Realistic Ge Concentration Profiles

The heterostructure profiles determined by APT and ToF‐SIMS describe Ge concentrations *C*(Ge) as a function of depth along the growth direction *z*, spatially averaging in the analyzed plane (*x*; *y*) at a given depth *z*. The analyzed area (*x*; *y*) depends on the method, APT or ToF‐SIMS. Note that the random nature of the SiGe alloy causes these profiles to vary spatially in plane, on an atomistic level, which in turn may cause fluctuations of the valley splitting. The mean and variance of the valley splitting both increase sensitively when the electron wavefunction overlaps strongly with Ge,^[^ ^37^ ^]^ as we will illustrate below. Since the valley splitting *E*_VS_ is determined by the alloy disorder in realistic heterostructures,^[^ ^37^ ^]^ increasing the electron wavefunction overlap with Ge (thus increasing the amount of alloy disorder) causes the average *E*_VS_ to grow. Given this sensitivity of the valley splitting and the fact that the typical confinement area of current experimentally implemented spin qubits approach the analysis area of APT, a series of statistically meaningful measurements of the valley splitting on a piece of a given heterostructure therefore, in principle, could provide a sensitive probe of the Ge concentration, even in the low‐Ge regime where the detection limit of APT measurements is reached. Up to now, such statistically meaningful samplings of the valley splitting have not been realized experimentally. Concerning the heterostructure studied here, a number of measurements were previously obtained in one device^[^ ^46^, ^51^ ^]^ from the same annealed heterostructure studied here, yielding values in the range of *E*_VS_ = 185‐212μeV, as the center position of the dot was shifted by 6 nm and, additionally, *E*_VS_ >100μeV estimated for the few other devices produced from that heterostructure. Below, we show that 1) these relatively high valley‐splitting values and their variations are consistent with theoretical predictions for the annealed sample, and 2) a non‐vanishing value of $C_{min}^{Ge}$ is crucial for obtaining the observed results. We also perform large‐scale valley‐splitting simulations to demonstrate how subtle features in the Ge concentration can have strong effects on the valley splitting.

To begin, we adopt the minimal 1D two‐band tight‐binding model of Boykin et al.^[^ ^26^ ^]^, which has been shown to quantitatively predict *E*_VS_ behavior in real devices.^[^ ^33^, ^37^ ^]^ To model alloy disorder in this 1D geometry, we start with the APT Ge concentration profiles of as‐grown and annealed samples fitted with Equations (2) and (3). We then introduce small random fluctuations in the Ge concentration for each atomic layer, consistent with random alloy disorder, following the approach of Ref. [37]. To build up a large statistical sample, we repeat this randomization many times and simulate *E*_VS_ for each case. We also compare the distributions of tight‐binding simulation results to effective‐mass theory, which predicts Rayleigh distributions for disorder‐dominated valley splittings.^[^ ^33^, ^37^ ^]^ More details on the theoretical tools used here are presented in the Supporting Information.

**Figure 4:**
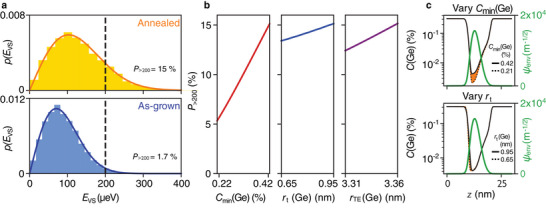
Valley splitting simulations of the as‐grown and post growth annealed quantum wells illustrate the impact of a small amount of Ge in the quantum well. a) Histograms show 10 000 simulations of *E*_VS_ for the annealed (top) and as‐grown (bottom) quantum‐well profiles, obtained using a minimal two‐band tight‐binding model.^[^ ^26^ ^]^ Each simulation represents a different realization of alloy disorder. Solid lines indicate the expected Rayleigh distribution for *E*_VS_, derived from effective‐mass theory.^[^ ^37^ ^]^ *P*_>200_ indicates the probability of obtaining *E*_VS_ larger than 200μeV (dashed line), as derived from these distributions. b) Starting with the annealed quantum well profile, we vary the minimum Ge concentration in the well, *C*_*min*_(Ge), from its value in the annealed well (*C*_*min*_(Ge) ≈ 0.42), to its value in the as‐grown well, (*C*_*min*_(Ge) ≈ 0.21), and we compute the resulting *P*_>200_ values (left, red). As the Ge content is reduced, so is *P*_>200_. We perform the same analysis with the top interface width *r*_*t*_(Ge) (center, blue) and the trailing edge width *r*_*TE*_(Ge) (right, purple). c) The annealed quantum well profile (black solid lines) is compared with two modified quantum wells (black dotted lines). Top: starting with the annealed well, the modified quantum well is found by reducing *C*_*min*_(Ge) from 0.42 to 0.21. Bottom: we reduce the top interface width *r*_*t*_(Ge) from 0.95 to 0.65 nm. In both plots, we highlight the Ge concentration differential in orange. We also include a simulation of the 1D quantum dot wavefunction envelope, ψ_*env*_ (green).

In **Figure** **4**a, we show histograms of 10000 1D tight‐binding simulations of *E*_VS_ for the annealed QW (top panel) and the as‐grown QW (bottom panel), where each iteration contains different random Ge concentration fluctuations consistent with alloy disorder, as described above. The solid lines show the corresponding Rayleigh distributions derived from the effective‐mass theory, using the respective QW parameters as inputs. We first confirm that the experimentally measured valley splittings, with typical values of 200μeV, are realistic and expected for this system. To do this, we compute the probability of finding valley splittings larger than this value, *P*_> 200_ = *P*(*E*_VS_ > 200μeV), for both of these distributions, where 200μeV is indicated in the figure by the dashed line. This analysis suggests that 200μeV is on the high side of the predicted distribution, but not unreasonably so. Moreover, we see that *P*_>200_ is more than eight times larger for the annealed sample than for the as‐grown sample, highlighting the strong dependence of the valley splitting on details of the Ge concentration profile and emphasizing the potential impact of sample processing on the valley splitting values determined in spin qubit experiments.

To illustrate this further, in Figure 4b we study the effect of three fitting parameters in Equations (2) and (3) on the valley splitting distribution: the minimum Ge concentration in the QW *C*_*min*_(Ge), the width of the top interface *r*_t_(Ge), and the trailing edge parameter *r*_TE_(Ge). In each case, to ensure physically reasonable results, we ramp a single parameter between its as‐grown and its post‐anneal values, while keeping the other parameters fixed at their values for the annealed samples. The effect of such variations on the Ge density profile is subtle, as illustrated in Figure 4c for the *C*_*min*_(Ge) and *r*_*t*_(Ge) parameters (note the logarithmic scale). Indeed, such variations approach the resolution limit of our fitting procedure (see Supporting Information). However, these variations significantly affect the valley splitting. Even slight, experimentally barely resolvable variations of *C*_*min*_(Ge) between 0.22 and 0.42% have a particularly strong effect, since they increase the Ge concentration in the region where the dot wavefunction is large, as shown in Figure 4c. Figure 4b illustrates that variations of *r*_T_ and *r*_*TE*_ yield smaller, but non‐negligible contributions. This analysis confirms the previous claims that, 1) for heterostructures without super‐sharp features (defined as features sharper than 2–3 atomic monolayers), fluctuations arising from alloy disorder dominate the valley splitting, and 2) this effect is enhanced when the wavefunction overlaps more strongly with the Ge.^[^ ^37^ ^]^ In the Supporting Information, we also show that experimentally observed variations of *E*_VS_ between 185 and 212μeV ^[^ ^46^ ^]^ are consistent with dot‐center shifts of 6 nm, for the *C*_*min*_(Ge) levels considered here.

## 4 Implications for Spin Qubits

Our tight‐binding and effective mass models, using the experimental APT Ge profiles as input, give strong indications that *E*_VS_ is dominated by alloy disorder in our heterostructure, as in others recently studied with APT^[^ ^33^, ^55^ ^]^ or scanning probe methods.^[^ ^38^ ^]^ Clearly, post‐growth annealing during the device fabrication, possibly enhanced by the presence of a slight segregation trailing edge at the bottom of the QW, seems to mostly be responsible for the experimental observation of valley splitting energies up to 212μeV ^[^ ^46^ ^]^ and >100μeV for the few other spin qubit devices experimentally tested in this heterostructure. Our study indicates that the ability to experimentally determine monolayer‐scale details in the Ge concentration QW profile of fabricated devices to capture post‐growth annealing effects, such as with APT, will be instrumental in providing input parameters for quantitative modeling of *E*_VS_, as well as for developing epitaxy recipes to maximize *E*_VS_. The knowledge of the top and bottom QW interface widths is not sufficient to capture the relevant effects. Any QW profile fit needs to be complemented by parameters reflecting the Ge environment of the wavefunction down to concentrations below 0.5%, as illustrated by the parameter $C_{min}\left(\mathrm{Ge}\right)=0.42$% in our study. Particularly noteworthy, the thinner the ^28^Si QW, the higher the probability that the wavefunction will overlap a Ge concentration relevant enough to boost *E*_VS_ in heterostructures with alloy disorder‐dominated interfaces, as shown in Figure 4c. Interestingly, recent reports on spin qubit devices fabricated in heterostructures with comparatively thin QWs frequently report experimentally determined *E*_VS_ > 100μeV.^[^ ^33^, ^34^, ^49^ ^]^

Our model predicts $P_{>100}=62$% for the annealed samples. Experimentally, we and the other recent reports on comparatively thin CVD grown QWs^[^ ^33^, ^34^, ^49^ ^]^ have found $E_{\mathrm{VS}}>100\;\mu eV$ in each measured quantum dot device. There are also reports where, in each measured qubit position, the valley splitting is smaller than $E_{\mathrm{VS}}<100\;\mu eV$.^[^ ^50^ ^]^ Although the amount of measured devices from each heterostructure is not sufficient to be statistically meaningful, these observations from different groups may hint that additional parameters are relevant for quantitative modeling, such as spatial correlations in the Ge concentration fluctuations and local strain in the QW.^[^ ^68^, ^69^ ^]^ Dislocation networks may produce such features (and also induce cross‐hatching of the surface). The ability to conduct experimental *E*_VS_ mappings in more extended quantum dot devices, such as electron shuttlers,^[^ ^50^ ^]^ should soon allow to further experimentally test the predicted variability of *E*_VS_ in heterostructures with alloy disorder‐dominated interfaces.

Regarding the dephasing times of the spin qubit, we obtained high and charge noise‐limited dephasing times $T_{2}^{*}\approx 20\;\mu s$,^[^ ^51^ ^]^ a record spin‐echo dephasing time $T_{2}^{\mathrm{echo}}=128\;\mu s$ ^[^ ^51^ ^]^ and an electron g‐factor of *g* = 2.00 ± 0.01^[^ ^46^ ^]^ for single spin qubits in our heterostructure. This indicates that neither the segregation‐induced, slightly delayed onset of Ge compared to natural Si at the top interface, nor the weak signatures of post‐growth annealing‐induced diffusion within the whole QW impact the spin qubit coherence in this ^28^Si QW. Our results thus suggest that small additions of Ge (below 0.5% in our heterostructure) may provide a balance between a sufficiently large valley‐splitting *E*_VS_ for spin qubit manipulation without introducing uncontrolled dephasing due to hyperfine interaction with ^73^Ge nuclear spins, which is much larger per atom than the one with ^29^Si^[^ ^70^ ^]^ nuclear spins. Anticipating further improvements in coherence times, isotope‐purified Ge could then be used in and around the ^28^Si QW ^[^ ^71^ ^]^, suppressing hyperfine interaction with ^73^Ge. Also, the fact that we determined *g* = 2.00 ± 0.01 ^[^ ^46^ ^]^ in this heterostructure, demonstrates that the slight addition of Ge to the ^28^Si QW does not induce relevant spin‐orbit coupling.

## 5 Conclusion

Summarizing our findings, our analysis allows us to experimentally extract realistic concentration profiles of the MBE heterostructure in its as‐grown state as well as after a post‐growth thermal anneal, representative of the thermal budget to which a spin qubit device is exposed during sample processing. We have found state of the art width values for the as‐grown interfaces between the ^28^Si QW and the top and bottom Si_0.7_Ge_0.3_ barrier. The post‐growth anneal leads to a seemingly small, but clearly detectable, broadening of the top interface due to isotropic bulk diffusion, while the bottom barrier width remains unchanged within the experimental detection limit. At the same time, we reveal signs of Ge segregation on comparably small length scales, imputable to the growth of the heterostructure. Segregation trailing edges have been reported on significantly larger length scales (due to the use of higher substrate temperatures during the epitaxy) before.^[^ ^61^ ^]^ APT proves here to be highly suited to analyze such rather subtle signatures of segregation and post‐growth annealing. In comparison, we found ToF‐SIMS to reveal the slightly retarded turn‐on of Ge at the top interface, but to be resolution‐limited regarding the trailing edge at the bottom interface and also the effect of post‐growth annealing. We attribute the resolution limitation to the heterostructure‐inherent strain relaxation. Using an APT needle in horizontal configuration to increase the analysis volume of the 10.5 nm thin QW, APT allowed us to assess an isotope purity of the ^28^Si QW of 50 ± 20ppm ^29^Si and down to the detection limit of 10ppm for ^30^Si. Additionally, we found the upper boundary of the Ge composition within the ^28^Si QW to be 0.3% in the horizontal needle configuration, which represents an unprecedented level of precision in a segregation or diffusion study in Si. This upper boundary agrees well with the parameter *C*_*min*_(Ge) of our APT Ge concentration profile fit function which increases from 0.21% in the as‐grown to 0.42% in the annealed QW and suggests that post‐growth annealing may have slightly increased the minimum Ge concentration present in the QW, presumably as a consequence of annealing‐induced post‐growth bulk diffusion.

Our theoretical model, which uses the fits to the experimental APT Ge concentration profiles as an input, provides strong indications that it is actually the post‐growth annealing, representative of the maximum thermal budget applied during quantum dot device processing, that is responsible for the experimental observation of large valley splitting energies for the spin qubits tested in this heterostructure. Indeed, the experimentally observed and comparably small increase of two fit function parameters under post‐growth annealing ‐ the width of the top barrier and a concentration offset suggesting a non‐zero minimum Ge concentration around 0.3% in the QW ‐ suffices to boost the probability to find *E*_VS_ >200μeV by more than a factor of 8 in the model. Our study strongly points out the importance of subtle Ge concentration changes in the direct environment of each qubit wavefunction. Notably, our results suggest that the risk of finding particularly low *E*_VS_ may be significantly reduced in qubit devices fabricated in comparatively thin QW heterostructures in the regime of alloy fluctuation‐dominated QW interfaces, in correlation with recent experimental studies.^[^ ^33^, ^34^, ^49^ ^]^

Hence, by employing the outstanding resolution limits of APT in Si/SiGe ‐ in terms of depth resolution and also composition resolution in a nanometer‐scale probe volume ‐ we show that being able to experimentally determine realistic concentration profiles down to the few‐monolayer‐limit and to concentrations <1% allows to resolve signatures of thin film growth‐inherent phenomena like Ge segregation or slight post‐growth anneal‐induced diffusion on such low length scales. Given that thin QWs, atomically sharp interfaces, delta‐like Ge spikes, sharp superlattices or the addition of a precise concentration of Ge to the QW are envisaged^[^ ^31^, ^35^, ^37^ ^]^ as an ingredient for massive scalability of spin qubits in Si/SiGe, empirical knowledge on such phenomena at realistic sample fabrication conditions will be key to develop viable novel heterostructure design approaches.

## 6 Experimental Section

### Molecular Beam Epitaxy

All heterostructures were grown in a solid source molecular beam epitaxy (MBE) ultra high vacuum chamber equipped with three independent electron beam evaporators for Si and Ge of natural isotopic composition and isotope‐purified ^28^Si with a 41 ppm residual composition of ^29^ *Si* determined in APT. At first, a relaxed Si_1 − *x*_Ge_*x*_ virtual substrate was grown on a Si(100) substrate without intentional miscut with natural isotopic composition and increasing Ge composition *x* up to a target composition of x=30%. The growth temperature for the virtual substrate was 500 °C. Next, 300 nm Si_0.7_Ge_0.3_ was grown followed by a nominal 12 nm strained QW layer of ^28^Si. The growth temperature for the QW was 350 °C with a deposition rate of 0.14 Å s^–1^. Finally, a 45 nm relaxed Si_0.7_Ge_0.3_ layer was grown, capped by nominally 1.5 nm of natural Si.

Post‐growth thermal treatment was done using a rapid thermal process (RTP) for 15 s at 700 °C with a 5 K s^–1^ ramp on a SiC carrier, referred to as the annealed heterostructure.

### Atom Probe Tomography

For atom probe tomography (APT), an additional 200 nm of electron beam evaporated amorphous silicon was deposited onto the SiGe heterostructure to prevent excessive damage during focused ion beam (FIB) processing. All APT measurements were performed on a Cameca local‐electrode atom‐probe (LEAP) 4000X‐Si system with a picosecond UV (wavelength of 355 nm) laser. The experimental conditions were given by a base temperature of 30 K, pulse repetition rate of 250 kHz, detection rate of 1% and laser energy of 30 pJ. The sample reconstruction was done using the software IVAS. The STEM analysis was performed on a TITAN (S)TEM from FEI using an accelerating voltage of 200 kV. The sample was rotated in edge on condition and viewed along the <110> direction. The HAADF‐STEM images were processed using Gatan's digital micrograph software.

**Figure 5:**
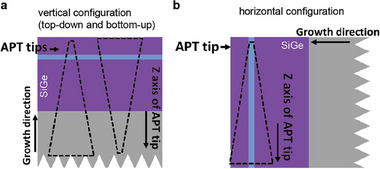
Schematic of the two tip geometries used for the APT analysis of the Si_0.7_Ge_0.3_/^28^Si/Si_0.7_Ge_0.3_ heterostructure: a) Vertical geometry. It delivers the tips for our two different analysis configurations for the isotope depth profiles in the QW, as highlighted by the dashed line: top–down (left) and bottom–up (right), see also Figure 1b. b) Horizontal geometry.

Prior to APT measurements, the needle‐shape specimens were prepared using the dual‐beam FIB system (FEI Helios NanoLab 650i) and annular Ga^+^ milling. As has been reported, interface aberrations occur during APT measurements for materials with changing evaporation fields.^[^ ^55^ ^]^ To infer these deviating effects, two specimen tips were prepared with opposing direction as schematically shown in **Figures** 5a and 1b to individually probe the top (bottom‐up specimen tip) and bottom (top‐down specimen tip) QW interface. Both were referred as the vertical needle configuration (Figure 5a). All specimen tips yield an approximate diameter of 100 nm. The 3D specimen reconstruction was calibrated with high resolution scanning transmission electron microscopy (HR STEM) measurements of the QW thickness in the prepared specimen tips.

**Figure 6:**
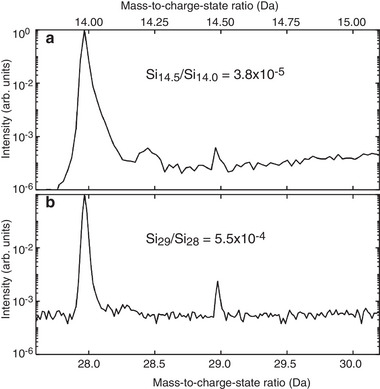
APT mass spectrum of the 99.9957 pure ^28^Si single crystal reference sample. a) Double charged Si ions peaks Si_14.5_ and Si_14_. b) Single charged Si ion peaks Si_29_ and Si_28_ mass peaks.

The sensitivity of APT was increased to spurious concentrations of Ge and Si isotopes in the QW by preparing a needle in a horizontal configuration (Figure 5b). This allowed to integrate a larger volume of atoms detected in the middle of the QW.^[^ ^72^ ^]^ A 50 ± 20ppm of residual ^29^Si in the geometric center of the QW was measured using mass‐to‐charge conversion based on single and double charged Si isotopes as explained below. In order to quantify the ^29^Si composition within the ^28^Si QW, a crystalline piece of the ^28^Si MBE source material (99.9957% ^28^Si single crystal with 41 ppm of ^29^Si^[^ ^73^ ^]^) was used as a reference sample. The mass spectra obtained for both, single and double charged Si isotopes, are shown in **Figure** **6**. Both spectra reveal a discrepancy between the ratios of Si_14.5_ over Si_14_ mass peaks (value is 3.8 × 10^−5^) and ratio of Si_29_ over Si_28_ mass peaks (value is 5.5 × 10^−4^). If the nominal ^29^Si over ^28^Si isotope concentration ratio in the 99.9957% pure ^28^Si single crystal was calculated, a value of 4.1 × 10^−5^ was obtained. This was almost the same as the value calculated for the Si_14.5_ over Si_14_ mass peak ratio in Figure 6a. Thus, this APT determined ratio was correct, while the Si_29_ peak was overestimated by APT due to the overlap with the SiH peak. Therefore, the correct ^29^Si composition determined by APT will be given by:

(1)

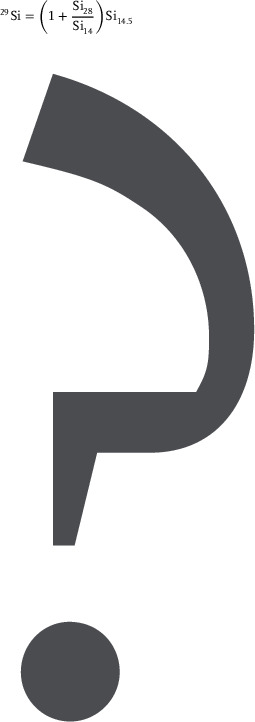

Applied to the APT data performed on the 99.9957 pure ^28^Si single crystal reference sample, this expression yields a value of 41 ± 10 ppm which fits exactly to previous measurements.^[^ ^73^ ^]^

### Time‐of‐Flight Secondary Ion Mass Spectrometry ToF‐SIMS

**Figure 7:**
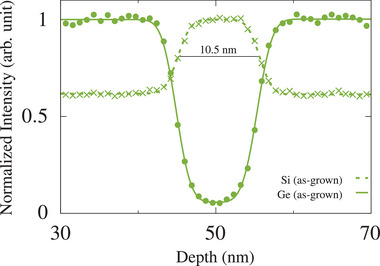
Normalized ToF‐SIMS intensities of ^28^Si and ^70, 72, 73, 74, 76^Ge (sum of all Ge isotopes) as a function of depth in the QW region (every 20th data point shown). By fitting Equation (2) to the profiles, the width of the QW was determined to be 10.5 ± 0.1 nm.

The SIMS experiments were performed using an ION‐ToF ToF.SIMS 5 system operating in dual beam mode. While a first ion beam (500 eV O_2_^+^, 40 nA) was sputtering a crater with a base area of 300μm×300μm, a second ion beam (15keV Bi_1_^+^, 0.25 pA) was progressively analyzing an area of 75μm×75μm in the center of the crater bottom. For optimum oxidation of the sample, oxygen flooding with a partial pressure of 1 × 10^−6^ mbar was used. The depths of the sputtered craters were determined with an uncertainty of about 10% using a Bruker DektakXT mechanical profilometer. **Figure** **7** shows the normalized intensities of ^28^Si and ^70, 72, 73, 74, 76^Ge (sum of all Ge isotopes) versus depth in the QW region. By fitting Equation (2) the width of the QW was determined to be 10.5 ± 0.1 nm.

### Fit Model for the Si and Ge Concentration at the Interfaces

For the analysis of the measured isotopic profiles, models based on error functions and sigmoid functions have been widely used.^[^ ^33^, ^55^, ^57^, ^58^, ^59^ ^]^ An error function based concentration profile *C*(*z*) was used in this work, to describe both heterostructure QW interfaces (the *top interface* and the *bottom interface*) for X = Si or Ge:

(2) $$\begin{array}{ccc} C\left(z;X\right)=\frac{C_{t}}{2}\cdot \left(\mathrm{erf}\left(-\frac{z-z_{t}}{r_{t}\left(X\right)}\right)+1\right) & + & \frac{C_{b}}{2}\cdot \left(\mathrm{erf}\left(\frac{z-z_{b}}{r_{b}\left(X\right)}\right)+1\right)\\ & & +C_{0}\left(X\right) \end{array}$$

where *z*_t_ and *z*_b_ are the positions of the top and bottom interface along the growth z‐axis (defined by the concentration profile of Ge), respectively. The corresponding interface width can be different in general and is given by *r*_t_ and *r*_b_, respectively. The concentration steps at the interfaces are given by *C*_*t*_ and *C*_*b*_ for the top and bottom interface, respectively. A constant offset concentrations of *C*_0_ was also taken into account. Note that *C*_0_ ⩽ *C*_min_, where *C*_min_ is the minimum concentration of the profile, depending on *C*_0_ but also on the broadening of the interfaces and the quantum well width. This model assumes a single constant parameter to describe the interface width. Depending upon the growth conditions, more complex profiles such as trailing edges can emerge, when Ge segregation affects the Ge incorporation rate at the heterostructure growth front. Therefore, the model was extened to capture such effects, which we dominantly observed for the bottom interface. It is suggested to use a non‐constant bottom interface width *r*_b_(*z*; *Ge*) in Equation (2):

(3) $$r_{b}\left(z;Ge\right)=\frac{r_{\mathrm{TE}}\left(\mathrm{Ge}\right)-r_{b}\left(\mathrm{Ge}\right)}{2}\cdot \left(\mathrm{erf}\left(-\frac{z-z_{b}-\varepsilon }{r_{\varepsilon }}\right)+1\right)+r_{b}\left(\mathrm{Ge}\right).$$

Here, we assume two regimes of different Ge incorporation rates split by a specific Ge concentration threshold *C*(*z*_b_ + ϵ; *Ge*) on the wafer surface during growth, where ϵ ≪ *r*_b_. For *C*(*z*; *Ge*) ≫ *C*(*z*_b_ + ϵ; *Ge*), the interface width is described by *r*_b_ equivalent to the model in Equation (2). For *C*(*z*; *Ge*) ≪ *C*(*z*_b_ + ϵ; *Ge*), the interface width is *r*_TE_ > *r*_b_ due to the decreased Ge incorporation rate. For changing Ge concentrations approaching *C*(*z*_b_ ± ϵ; *Ge*), a Gaussian transition was assumed from *r*_b_(Ge) to *r*_TE_(Ge) or vice versa within 0 ⩽ *r*_ϵ_ ⩽ 2.5 nm. The latter limit was used to ensure smooth transitions between both regimes and corresponds to the width of the assumed Gaussian across the overall wafer.

Finally, a comparison of the interface width parameters *r*_t_ and *r*_b_ of the error function model with the sigmoid model used in the literature for recent analyses of CVD grown heterostructures is provided^[^ ^33^, ^55^ ^]^:

(4) $$\frac{2}{1+e^{-\left(z-z_{0}\right)/\tau }}-1\approx \frac{x-z_{0}}{2\tau }+O\left({\left(\frac{x-z_{0}}{2\tau }\right)}^{3}\right)$$

where *z* is the position along the growth direction, *z*_0_ is the position of the material interface and τ is the interface diffusion length in the sigmoid model.^[^ ^33^, ^55^ ^]^ The used error function model for a single material interface is described by

(5) $$\mathrm{erf}\left(\frac{x-z_{0}}{r}\right)\approx \frac{2}{\sqrt{\pi }}\frac{x-z_{0}}{r}+O\left({\left(\frac{x-z_{0}}{r}\right)}^{3}\right)$$

Hence, the relation between the interface widths respectively defined in both models is $4\tau \approx \sqrt{\pi }r$. This relation was used for making comparisons in the table summarizing all measured APT needles in the Supporting Information.

## Conflict of Interest

The authors declare no conflict of interest.

## Supporting information

Supporting Information

## Data Availability

The data that support the findings of this study are available from the corresponding author upon reasonable request.

## References

[1] S. M. Frolov , M. J. Manfra , J. D. Sau , Nat. Phys. 2020, 16, 718.

[2] C. E. Murray , Mater. Sci. Eng. R Rep 2021, 146, 100646.

[3] A. Premkumar , C. Weiland , S. Hwang , B. Jäck , A. P. M. Place , I. Waluyo , A. Hunt , V. Bisogni , J. Pelliciari , A. Barbour , M. S. Miller , P. Russo , F. Camino , K. Kisslinger , X. Tong , M. S. Hybertsen , A. A. Houck , I. Jarrige , Commun. Mater. 2021, 2, 72.

[4] A. P. M. Place , L. V. Rodgers , P. Mundada , B. M. Smitham , M. Fitzpatrick , Z. Leng , A. Premkumar , J. Bryon , A. Vrajitoarea , S. Sussman , G. Cheng , T. Madhavan , H. K. Babla , X. H. Le , Y. Gang , B. Jäck , A. Gyenis , N. Yao , R. J. Cava , N. P. de Leon , A. A. Houck , Nat. Commun. 2021, 12, 1779. 33741989 10.1038/s41467-021-22030-5 PMC7979772

[5] S. G. J. Philips , M. T. Mądzik , S. V. Amitonov , S. L. de Snoo , M. Russ , N. Kalhor , C. Volk , W. I. L. Lawrie , D. Brousse , L. Tryputen , B. P. Wuetz , A. Sammak , M. Veldhorst , G. Scappucci , L. M. K. Vandersypen , Nature 2022, 609, 919. 36171383 10.1038/s41586-022-05117-x PMC9519456

[6] S. Neyens , O. K. Zietz , T. F. Watson , F. Luthi , A. Nethwewala , H. C. George , E. Henry , M. Islam , A. J. Wagner , F. Borjans , E. J. Connors , J. Corrigan , M. J. Curry , D. Keith , R. Kotlyar , L. F. Lampert , M. T. Mądzik , K. Millard , F. A. Mohiyaddin , S. Pellerano , R. Pillarisetty , M. Ramsey , R. Savytskyy , S. Schaal , G. Zheng , J. Ziegler , N. C. Bishop , S. Bojarski , J. Roberts , J. S. Clarke , et al., Nature 2024, 629, 80. 38693414 10.1038/s41586-024-07275-6 PMC11062914

[7] A. Wild , J. Kierig , J. Sailer , J. W. Ager , E. Haller , G. Abstreiter , S. Ludwig , D. Bougeard , Appl. Phys. Lett. 2012, 100, 143110.

[8] F. A. Zwanenburg , A. S. Dzurak , A. Morello , M. Y. Simmons , L. C. L. Hollenberg , G. Klimeck , S. Rogge , S. N. Coppersmith , M. A. Eriksson , Rev. of Mod. Phys. 2013, 85, 961.

[9] J. Yoneda , K. Takeda , T. Otsuka , T. Nakajima , M. R. Delbecq , G. Allison , T. Honda , T. Kodera , S. Oda , Y. Hoshi , N. Usami , K. M. Itoh , S. Tarucha , Nat. Nanotechnol. 2018, 13, 102. 29255292 10.1038/s41565-017-0014-x

[10] T. Struck , J. Lindner , A. Hollmann , F. Schauer , A. Schmidbauer , D. Bougeard , L. R. Schreiber , Sci. Rep. 2021, 11, 16203. 34376730 10.1038/s41598-021-95562-x PMC8355192

[11] E. Kammerloher , A. Schmidbauer , L. Diebel , I. Seidler , M. Neul , M. Künne , A. Ludwig , J. Ritzmann , A. Wieck , D. Bougeard , L. R. Schreiber , H. Bluhm , Phys. Rev. Appl. 2024, 22, 024044.

[12] A. Noiri , K. Takeda , T. Nakajima , T. Kobayashi , A. Sammak , G. Scappucci , S. Tarucha , Nature 2022, 601, 338. 35046603 10.1038/s41586-021-04182-y

[13] A. R. Mills , C. R. Guinn , M. J. Gullans , A. J. Sigillito , M. M. Feldman , E. Nielsen , J. R. Petta , Sci. Adv. 2022, 8, eabn5130. 35385308 10.1126/sciadv.abn5130 PMC8986105

[14] X. Xue , M. Russ , N. Samkharadze , B. Undseth , A. Sammak , G. Scappucci , L. M. K. Vandersypen , Nature 2022, 601, 343. 35046604 10.1038/s41586-021-04273-w PMC8770146

[15] L. M. K. Vandersypen , H. Bluhm , J. S. Clarke , A. S. Dzurak , R. Ishihara , A. Morello , D. J. Reilly , L. R. Schreiber , M. Veldhorst , npj Quantum Inf. 2017, 3, 34.

[16] I. Seidler , T. Struck , R. Xue , N. Focke , S. Trellenkamp , H. Bluhm , L. R. Schreiber , npj Quantum Inf. 2022, 8, 100.

[17] R. Xue , M. Beer , I. Seidler , S. Humpohl , J. Tu , T. Stefan , T. Struck , H. Bluhm , L. R. Schreiber , Nat. Commun. 2024, 15, 2296. 38485971 10.1038/s41467-024-46519-x PMC10940717

[18] T. Struck , M. Volmer , L. Visser , T. Offermann , R. Xue , J.‐S. Tu , S. Trellenkamp , Ł. Cywiński , H. Bluhm , L. R. Schreiber , Nat. Commun. 2024, 15, 1325. 38351007 10.1038/s41467-024-45583-7 PMC10864332

[19] V. Langrock , J. A. Krzywda , N. Focke , I. Seidler , L. R. Schreiber , Ł. Cywiński , PRX Quantum 2023, 4, 020305.

[20] M. Künne , A. Willmes , M. Oberländer , C. Gorjaew , J. D. Teske , H. Bhardwaj , M. Beer , E. Kammerloher , R. Otten , I. Seidler , R. Xue , L. R. Schreiber , H. Bluhm , Nat. Commun. 2024, 15, 4977. 38862531 10.1038/s41467-024-49182-4 PMC11166970

[21] L. V. C. Assali , H. M. Petrilli , R. B. Capaz , B. Koiller , X. Hu , S. Das Sarma , Phys. Rev. B 2011, 83, 165301.

[22] M. Neul , I. V. Sprave , L. K. Diebel , L. G. Zinkl , F. Fuchs , Y. Yamamoto , C. Vedder , D. Bougeard , L. R. Schreiber , Phys. Rev. Mater. 2024, 8, 043801.

[23] R. Ferdous , E. Kawakami , P. Scarlino , M. P. Nowak , D. Ward , D. Savage , M. Lagally , S. Coppersmith , M. Friesen , M. A. Eriksson , L. M. K. Vandersypen , npj Quantum Inf. 2018, 4, 26.

[24] M. P. Losert , M. Oberländer , J. D. Teske , M. Volmer , L. R. Schreiber , H. Bluhm , S. N. Coppersmith , M. Friesen , arXiv:2405.01832 2024.

[25] T. Ando , Physical Review B 1979, 19, 3089.

[26] T. B. Boykin , G. Klimeck , M. A. Eriksson , M. Friesen , S. N. Coppersmith , P. von Allmen , F. Oyafuso , S. Lee , Appl. Phys. Lett. 2004, 84, 115.

[27] M. Friesen , M. A. Eriksson , S. N. Coppersmith , Appl. Phys. Lett. 2006, 89, 202106.

[28] M. Friesen , S. Chutia , C. Tahan , S. N. Coppersmith , Phys. Rev. B 2007, 75, 115318.

[29] D. Culcer , X. Hu , S. Das Sarma , Phys. Rev. B 2010, 82, 205315.

[30] Z. Shi , C. B. Simmons , J. R. Prance , J. King Gamble , M. Friesen , D. E. Savage , M. G. Lagally , S. N. Coppersmith , M. A. Eriksson , Appl. Phys. Lett. 2011, 99, 233108.

[31] L. Zhang , J.‐W. Luo , A. Saraiva , B. Koiller , A. Zunger , Nat. Commun. 2013, 4, 2396. 24013452 10.1038/ncomms3396 PMC3778719

[32] A. Hosseinkhani , G. Burkard , Phys. Rev. Res. 2020, 2, 043180.

[33] B. P. Wuetz , M. P. Losert , S. Koelling , L. E. A. Stehouwer , A.‐M. J. Zwerver , S. G. J. Philips , M. T. Mądzik , X. Xue , G. Zheng , M. Lodari , S. V. Amitonov , N. Samkharadze , A. Sammak , L. M. K. Vandersypen , R. Rahman , S. N. Coppersmith , O. Moutanabbir , M. Friesen , G. Scappucci , Nat. Commun. 2022, 13, 7730. 36513678 10.1038/s41467-022-35458-0 PMC9747794

[34] E. H. Chen , K. Raach , A. Pan , A. A. Kiselev , E. Acuna , J. Z. Blumoff , T. Brecht , M. D. Choi , W. Ha , D. R. Hulbert , M. P. Jura , T. E. Keating , R. Noah , B. Sun , B. J. Thomas , M. G. Borselli , C. Jackson , M. T. Rakher , R. S. Ross , Phys. Rev. Appl. 2021, 15, 044033.

[35] G. Wang , Z.‐G. Song , J.‐W. Luo , S.‐S. Li , Phys. Rev. B 2022, 105, 165308.

[36] J. P. Dodson , H. E. Ercan , J. Corrigan , M. P. Losert , N. Holman , T. McJunkin , L. F. Edge , M. Friesen , S. N. Coppersmith , M. A. Eriksson , Phys. Rev. Lett. 2022, 128, 146802. 35476478 10.1103/PhysRevLett.128.146802

[37] M. P. Losert , M. A. Eriksson , R. Joynt , R. Rahman , G. Scappucci , S. N. Coppersmith , M. Friesen , Phys. Rev. B 2023, 108, 125405.

[38] L. F. Peña , J. C. Koepke , J. H. Dycus , A. Mounce , A. D. Baczewski , N. T. Jacobson , E. Bussmann , npj Quantum Inf. 2024, 10, 33.

[39] M. G. Borselli , R. S. Ross , A. A. Kiselev , E. T. Croke , K. S. Holabird , P. W. Deelman , L. D. Warren , I. Alvarado‐Rodriguez , I. Milosavljevic , F. C. Ku , W. S. Wong , A. E. Schmitz , M. Sokolich , M. F. Gyure , A. T. Hunter , Appl. Phys. Lett. 2011, 98, 123118.

[40] E. Kawakami , P. Scarlino , D. R. Ward , F. R. Braakman , D. E. Savage , M. G. Lagally , M. Friesen , S. N. Coppersmith , M. A. Eriksson , L. M. K. Vandersypen , Nat. Nanotechnol. 2014, 9, 666. 25108810 10.1038/nnano.2014.153

[41] P. Scarlino , E. Kawakami , T. Jullien , D. R. Ward , D. E. Savage , M. G. Lagally , M. Friesen , S. N. Coppersmith , M. A. Eriksson , L. M. K. Vandersypen , Phys. Rev. B 2017, 95, 165429. 10.1103/PhysRevLett.115.106802 26382693

[42] D. M. Zajac , T. M. Hazard , X. Mi , K. Wang , J. R. Petta , Appl. Phys. Lett. 2015, 106, 223507.

[43] X. Mi , C. G. Péterfalvi , G. Burkard , J. R. Petta , Phys. Rev. Lett. 2017, 119, 176803. 29219471 10.1103/PhysRevLett.119.176803

[44] T. F. Watson , S. G. J. Philips , E. Kawakami , D. R. Ward , P. Scarlino , M. Veldhorst , D. E. Savage , M. G. Lagally , M. Friesen , S. N. Coppersmith , M. A. Eriksson , L. M. K. Vandersypen , Nature 2018, 555, 633. 29443962 10.1038/nature25766

[45] F. Borjans , D. M. Zajac , T. M. Hazard , J. R. Petta , Phys. Rev. Appl. 2019, 11, 044063.

[46] A. Hollmann , T. Struck , V. Langrock , A. Schmidbauer , F. Schauer , T. Leonhardt , K. Sawano , H. Riemann , N. V. Abrosimov , D. Bougeard , L. R. Schreiber , Phys. Rev. Appl. 2020, 13, 034068.

[47] T. McJunkin , E. R. MacQuarrie , L. Tom , S. F. Neyens , J. P. Dodson , B. Thorgrimsson , J. Corrigan , H. E. Ercan , D. E. Savage , M. G. Lagally , R. Joynt , S. N. Coppersmith , M. Friesen , M. A. Eriksson , Phys. Rev. B 2021, 104, 085406. 10.1103/PhysRevLett.127.127701 34597063

[48] X. Mi , M. Benito , S. Putz , D. M. Zajac , J. M. Taylor , G. Burkard , J. R. Petta , Nature 2018, 555, 599. 29443961 10.1038/nature25769

[49] D. Degli Esposti , L. E. A. Stehouwer , O. Gül , N. Samkharadze , C. Déprez , M. Meyer , I. N. Meijer , L. Tryputen , S. Karwal , M. Botifoll , J. Arbiol , S. V. Amitonov , L. M. K. Vandersypen , A. Sammak , M. Veldhorst , G. Scappucci , npj Quantum Inf. 2024, 10, 32.

[50] M. Volmer , T. Struck , A. Sala , B. Chen , M. Oberländer , T. Offermann , R. Xue , L. Visser , J.‐S. Tu , S. Trellenkamp , Łukasz Cywińki , H. Bluhm , L. R. Schreiber , npj Quantum Inf. 2024, 10, 61.

[51] T. Struck , A. Hollmann , F. Schauer , O. Fedorets , A. Schmidbauer , K. Sawano , H. Riemann , N. V. Abrosimov , Ł. Cywińki , D. Bougeard , L. R. Schreiber , npj Quantum Inf. 2020, 6, 2056.

[52] F. Vurpillot , D. Larson , A. Cerezo , Surf. Interface Anal. 2004, 36, 552.

[53] C. Fletcher , M. P. Moody , D. Haley , J. Phys. D: Appl. Phys. 2020, 53, 475303.

[54] S. Koelling , M. Gilbert , J. Goossens , A. Hikavyy , O. Richard , W. Vandervorst , Appl. Phys. Lett. 2009, 95, 144106.

[55] O. Dyck , D. N. Leonard , L. F. Edge , C. A. Jackson , E. J. Pritchett , P. W. Deelman , J. D. Poplawsky , Adv. Mater. Interfaces 2017, 4, 1700622.

[56] S. Koelling , L. E. A. Stehouwer , B. Paquelet Wuetz , G. Scappucci , O. Moutanabbir , Adv. Mater. Interfaces 2023, 10, 2201189.

[57] T. Südkamp , H. Bracht , Phys. Rev. B 2016, 94, 125208.

[58] R. Kube , H. Bracht , J. L. Hansen , A. N. Larsen , E. E. Haller , S. Paul , W. Lerch , J. Appl. Phys. 2010, 107, 073520.

[59] R. Kube , H. Bracht , E. Hüger , H. Schmidt , J. L. Hansen , A. N. Larsen , J. W. Ager III , E. E. Haller , T. Geue , J. Stahn , Phys. Rev. B 2013, 88, 085206.

[60] D. J. Godbey , M. G. Ancona , Appl. Phys. Lett. 1992, 61, 2217.

[61] S. Fukatsu , K. Fujita , H. Yaguchi , Y. Shiraki , R. Ito , Appl. Phys. Lett. 1991, 59, 2103.

[62] J. Tröger , R. Kersting , B. Hagenhoff , D. Bougeard , N. V. Abrosimov , J. Klos , L. R. Schreiber , H. Bracht , arXiv:2407.17985 1994, 65, 711.

[63] D. J. Godbey , J. V. Lill , J. Deppe , K. D. Hobart , Appl. Phys. Lett. 1994, 65, 711.

[64] D. Godbey , M. Ancona , Surf. Sci. 1998, 395, 60.

[65] G. G. Jernigan , P. E. Thompson , C. L. Silvestre , Appl. Phys. Lett. 1996, 69, 1894.

[66] G. G. Jernigan , P. E. Thompson , C. L. Silvestre , Surf. Sci. 1997, 380, 417.

[67] A. Pramann , O. Rienitz , D. Schiel , J. Schlote , B. Güttler , S. Valkiers , Metrologia 2011, 48, 20.

[68] C. Corley‐Wiciak , M. H. Zoellner , I. Zaitsev , K. Anand , E. Zatterin , Y. Yamamoto , A. A. Corley‐Wiciak , F. Reichmann , W. Langheinrich , L. R. Schreiber , C. L. Manganelli , M. Virgilio , C. Richter , G. Capellini , Phys. Rev. Appl. 2023, 20, 024056.

[69] C. Corley‐Wiciak , C. Richter , M. H. Zoellner , I. Zaitsev , C. L. Manganelli , E. Zatterin , T. U. Schülli , A. A. Corley‐Wiciak , J. Katzer , F. Reichmann , W. M. Klesse , N. W. Hendrickx , A. Sammak , M. Veldhorst , G. Scappucci , M. Virgilio , G. Capellini , ACS Appl. Mater. Interfaces 2023, 15, 3119. 36598897 10.1021/acsami.2c17395 PMC9869329

[70] D. K. Wilson , Phys. Rev. 1964, 134, A265.

[71] J. Sailer , V. Lang , G. Abstreiter , G. Tsuchiya , K. M. Itoh , J. W. Ager III , E. E. Haller , D. Kupidura , D. Harbusch , S. Ludwig , D. Bougeard , Phys. Status Solidi RRL 2009, 3, 61.

[72] O. Cojocaru‐Mirédin , T. Schwarz , P.‐P. Choi , M. Herbig , R. Wuerz , D. Raabe , J. Vis. Exp. 2013, 74, e50376. 10.3791/50376 PMC3667468 23629452

[73] P. Becker , H.‐J. Pohl , H. Riemann , N. Abrosimov , Phys. Status Solidi A 2010, 207, 49.

