# Supplementary material for: Atomistic Compositional Details and Their Importance for Spin Qubits in Isotope‐Purified Silicon Quantum Wells
Source: Adv Sci (Weinh). 2024 Sep 11;11(42):2407442. doi: 10.1002/advs.202407442 (PMC11558133; doi:10.1002/advs.202407442)
Supplement: Supplementary file 1 — Supporting Information [file ADVS-11-2407442-s001.pdf]

ADVANCED SCIENCE
Open Access

Supporting Information

for *Adv. Sci.*, DOI 10.1002/advs.202407442

Atomistic Compositional Details and Their Importance for Spin Qubits in Isotope-Purified Silicon Quantum Wells

Jan Klos, Jan Tröger, Jens Keutgen, Merritt P. Losert, Nikolay V. Abrosimov, Joachim Knoch, Hartmut Bracht, Susan N. Coppersmith, Mark Friesen, Oana Cojocaru-Mirédin, Lars R. Schreiber* and Dominique Bougeard**

Supporting Information: Atomistic compositional details and their importance for spin qubits in isotope-purified silicon quantum wells

J. Klos,† J. Tröger,‡ J. Keutgen,¶ M. P. Losert,§ N. V. Abrosimov,‖ J. Knoch,⊥ H. Bracht,‡ S. N. Coppersmith,# M. Friesen,§ O. Cojocaru-Mirédin,¶,@ L. R. Schreiber,*,†,△ and D. Bougeard*,∇

†*JARA-FIT Institute for Quantum Information, Forschungszentrum Jülich GmbH & RWTH Aachen University, Aachen, Germany*

‡*Institute of Materials Physics, University of Münster, Münster, Germany*

¶*I. Physikalisches Institut IA, RWTH Aachen University, Aachen, Germany*

§*University of Wisconsin-Madison, Madison, Wisconsin, USA*

‖*Leibniz-Institut für Kristallzüchtung (IKZ), Berlin, Germany*

⊥*Institute of Semiconductor Electronics, RWTH Aachen University, Aachen, Germany*

#*University of New South Wales, Sydney, Australia*

@*INATECH, Albert-Ludwigs Universität Freiburg, Freiburg im Breisgau, Germany*

△*ARQUE Systems GmbH, Aachen, Germany*

∇*Institut für Experimentelle und Angewandte Physik, Universität Regensburg, Regensburg, Germany*

E-mail: lars.schreiber@physik.rwth-aachen.de; dominique.bougeard@ur.de

Atomic Force Microscopy of the ToF-SIMS crater

A Park Systems XE-100 Atomic Force Microscope was used to analyze the topography in the crater resulting from the ToF-SIMS measurements. Using a NSC15 tip in non-contact mode, an area of 8.0 µm × 8.0 µm in the sputter crater was imaged. The collected data, presented in Figure S1 show a regular terracing on a µm scale in two perpendicular crystal directions. This is known as surface corrugation[1] or cross-hatching and results from the strain relaxation via dislocations of a SiGe layer on a Si substrate. The roughness was determined to be 3.2 nm (RMS). As discussed in the main text, this roughness limits the depth resolution of the ToF-SIMS analysis.

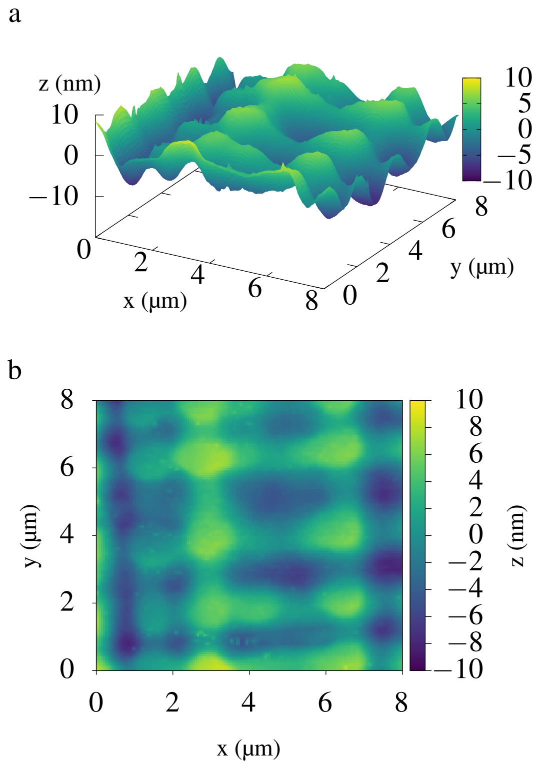

Figure S1: Topography of a 8.0 µm × 8.0 µm area in the ToF-SIMS crater measured by means of atomic force microscopy. (a) three-dimensional representation, (b) two-dimensional representation. The regular terracing indicates surface corrugation resulting from strain relaxation via the formation of dislocations.[1]

Simulation of valley splitting

To study valley splitting statistics in the heterostructures derived from APT fits, we use the 1D two-band tight-binding model of Boykin et al.[2] Each cell in this model corresponds to one mono-atomic layer of width $a_0/4$, where $a_0 = 0.543\,\mathrm{nm}$ is the Si lattice constant. This model contains a nearest-neighbor hopping term $t_1 = 0.68$ eV and a next-nearest-neighbor hopping term $t_2 = 0.61$ eV, allowing it to replicate the position and curvature of the z-valley minima of the conduction-band structure in Si. The potential energy arising from a vertical electric field is given by $U_z = eE_z z$, where we set $E_z = 1.34\,\mathrm{mV/nm}$, as consistent with Ref. 3. The quantum well potential energy is given by

$$U_{\mathrm{qw}}(z) = \Delta E_c \frac{x(z) - x_w}{x_s - x_w}, \quad (1)$$

where ΔE_c is the conduction band offset, $x(z)$ is the local Si concentration at position z, x_w is the maximum Si concentration in the quantum well, and x_s is the Si concentration in the SiGe barrier/substrate. The conduction band offset is computed following Refs. 4 and 5:

$$\Delta E_c = (x_w - x_s)\left[\frac{x_w}{1 - x_s}\Delta E^{\mathrm{Si}}_{\Delta_2}(x_s) - \frac{1 - x_w}{x_s}\Delta E^{\mathrm{Ge}}_{\Delta_2}(x_s)\right], \quad (2)$$

where $\Delta E^{\mathrm{Si/Ge}}_{\Delta_2}(x)$ are the Δ_2 conduction band offsets of fully strained Si/Ge grown epitaxiallhy on an unstrained $\mathrm{Si}_x\mathrm{Ge}_{1-x}$ substrate, approximately given by

$$\Delta E^{\mathrm{Si}}_{\Delta_2}(x) \approx -0.502(1 - x)\ (\mathrm{eV}), \quad (3)$$

$$\Delta E^{\mathrm{Ge}}_{\Delta_2}(x) \approx 0.743 - 0.625(1 - x)\ (\mathrm{eV}). \quad (4)$$

Both U_z and U_{qw} are included as onsite parameters in the tight-binding model. To add alloy disorder to our model, we allow random fluctuations in the Si concentration at each layer. Following Refs. 4 and 5, the concentration fluctuation at each layer is given by $\delta_z \sim \frac{1}{N_{\mathrm{eff}}}\mathrm{Binom}(N_{\mathrm{eff}}, \bar{x}_z)$, where $\mathrm{Binom}(n, p)$ is the binomial distribution of n trials with suc-

cess probability p, $N_{\text{eff}} = 4\pi a_{\text{dot}}^2/a_0^2$ and $\bar{x}_z$ is the mean Si concentration at position z, defined by the APT fits. The characteristic dot size $a_{\text{dot}} = \sqrt{\hbar/m_t\omega_{\text{orb}}}$, where $m_t = 0.19m_e$ is the transverse effective mass of Si and $\hbar\omega_{\text{orb}}$ is the characteristic energy splitting of the lateral, quantum-dot confinement potential. For simplicity, we assume an isotropic parabolic confinement potential with characteristic orbital energy splitting of $\hbar\omega_{\text{orb}} = 2.5\,\text{meV}$, consistent with measurements performed on this device.[3]

From effective mass theory,[5] we expect the valley splittings for the QWs considered in this work to follow the Rayleigh probability distribution $f_{\text{Rayleigh}}(E_{\text{VS}}) = (E_{\text{VS}}/s^2)\exp(-E_{\text{VS}}^2/2s^2)$. The scale parameter s is related to the mean valley splitting $\overline{E_{\text{VS}}}$ by $\overline{E_{\text{VS}}} = s\sqrt{\pi/2}$. From effective mass theory, the mean valley splitting is given by $\overline{E_{\text{VS}}} = \sqrt{\pi}\sigma_\Delta$, where

$$\sigma_\Delta^2 = \frac{1}{\pi}\left[\frac{a_0^2\Delta E_c}{8a_{\text{dot}}(x_w - x_s)}\right]^2 \sum_z |\psi_{\text{env}}(z)|^4 \bar{x}_z(1-\bar{x}_z). \tag{5}$$

Here, the parameter σ_Δ^2 is the variance of the inter-valley coupling Δ, where $E_{\text{VS}} = 2|\Delta|$. The envelope function ψ_{env} is computed by solving a discretized Schrödinger equation for a given quantum well potential, ignoring valley physics. Using the fits to the concentration profiles experimentally obtained in APT, we can compute σ_Δ^2 from Eq. (5), which is then used to define the expected Rayleigh distribution, as plotted in Figure 4(a) of the main text. The probabilities $P_{>200} = P(E_{\text{VS}} > 200\,\mu\text{eV})$, as reported in Figure 4 of the main text, can be computed from the resulting Rayleigh distributions.

We can also examine the valley splitting behavior as a dot moves across a heterostructure, using effective mass theory. Here, we define $\Delta_1 = r_1 e^{i\theta_1}$ and $\Delta_2 = r_2 e^{i\theta_2}$ as the intervalley couplings of a dot at two locations in the xy-plane, separated by a distance d. Previous theoretical work[5] has shown that the joint probability density function for Δ_1 and Δ_2 is given by

$$p(\Delta_1, \Delta_2) = \frac{1}{\sqrt{(2\pi)^4|\boldsymbol{\Sigma}|}}\exp\left(-\frac{1}{2}\mathbf{v}^T\boldsymbol{\Sigma}^{-1}\mathbf{v}\right), \tag{6}$$

where the basis vector is defined as $\mathbf{v} = \{\text{Re}\Delta_1, \text{Im}\Delta_1, \text{Re}\Delta_2, \text{Im}\Delta_2\}$ and the covariance

matrix is given by

$$\mathbf{\Sigma} = \frac{\sigma_\Delta^2}{2} \begin{pmatrix} 1 & 0 & A & 0 \\ 0 & 1 & 0 & A \\ A & 0 & 1 & 0 \\ 0 & A & 0 & 1 \end{pmatrix}, \tag{7}$$

with $A = \exp(-d^2/2a_{\text{dot}}^2)$. Expanding the matrix product and the determinant, changing variables to r_i and θ_i, and performing the angular integration yields

$$p(r_1, r_2) = \frac{4r_1 r_2}{\sigma_\Delta^4} \frac{\exp\left[-\frac{(r_1^2+r_2^2)e^{d^2/a_{\text{dot}}^2}}{\left(e^{d^2/a_{\text{dot}}^2}-1\right)\sigma_\Delta^2}\right]}{\sqrt{1+e^{-2d^2/a_{\text{dot}}^2} - 2e^{-d^2/a_{\text{dot}}^2}}} I_0\left(\frac{r_1 r_2}{\sigma_\Delta^2}\text{csch}(d^2/2a_{\text{dot}}^2)\right), \tag{8}$$

where $I_0(x)$ is a modified Bessel function of the first kind. If we substitute $E_{\text{VS},i} = 2r_i$, Eq. (8) then gives the joint probability density function for two valley splitting measurements, separated by distance d in the heterostructure.

In previously published work,[3] experimental measurements showed variations of E_{VS} between 185 µeV and 212 µeV when shifting the quantum dot laterally by 6 nm. We can use Eq. (8) to analyze the likelihood of such a shift. Figure S2 illustrates the 10, 50, and 90 percentile contours of the joint probability density function for two valley splitting measurements, separated laterally by 6 nm, for the annealed QW [red contours, $C_{min}(Ge) \approx 0.42\,\%$] and a slightly modified annealed QW, where we modify only the parameter $C_0(Ge)$ such that the minimum Ge concentration in the QW is zero [blue contours, $C_{min}(Ge) = 0\,\%$]. The black diamond in Figure S2 indicates the experimental measurements, $E_{\text{VS},1} = 212$ µeV and $E_{\text{VS},2} = 185$ µeV. For the annealed QW, these measurements fall within the 90 percentile contour, indicating that they are not unlikely. However, if the minimum Ge concentration in the QW was zero, these measurements would lie well outside the 90 percentile contour, indicating that they are very unlikely. This analysis further supports the claim that a small, nonzero Ge concentration inside the QW provides results consistent with the large valley splittings measured in Ref. 3.

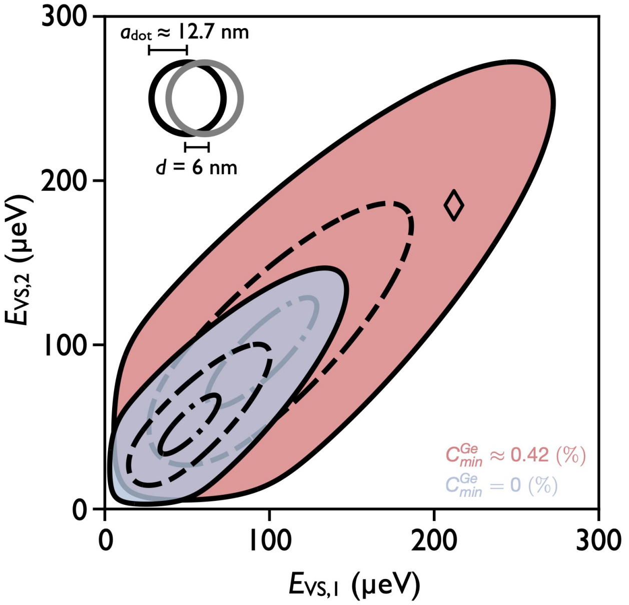

Figure S2: The joint probability distributions, defined in Eq. (8), for two valley splitting measurements, $E_{\mathrm{VS},1}$ and $E_{\mathrm{VS},2}$, performed for dot positions separated by 6 nm. The shaded areas indicate the 90 percentile regions of the joint pdf for the annealed QW (red, $C_{min}(Ge) \approx 0.4\,\%$) and an annealed QW with a fully depleted $C_{min}(Ge)$ (blue, $C_{min}(Ge) = 0\,\%$; see Supporting Information text for explanation). Dashed and dot-dashed lines indicate the 50 and 10 percentile regions, respectively. The inset indicates the motion of the dot, relative to the dot radius a_{dot}.

Fit parameters obtained from eight APT specimen

In total, we measured eight APT specimen in the vertical direction (see Figure 5a of the main text for the definition of the geometry). In Table S1 and Table S2, we provide the detail of all relevant fit parameters for each of the measured specimen for completeness. Four specimen were annealed (fitted data reported in Table S1). The other four specimen were measured as grown (fitted data reported in Table S2). In both tables we distinguish between *Top interface* (SiGe/^{28}Si QW top interface) and *Bottom interface* (^{28}Si/SiGe QW bottom interface). For each QW interface two specimen tips were measured in the most sensitive analysis tip direction for that specific interface (see Figure 5a of the main text for the definition of the geometry). The data of the two specimen are labelled 1 & 2 for each interface in the tables.

For each specimen Table S1 and Table S2 report the values obtained from the fits to the experimental data of *Si* (standing here for the sum of the composition in the isotopes ^{29,30}Si) and for *Ge* (standing for the sum of the composition in the isotopes ^{70,72,73,74,76}Ge). The Ge bottom interface was fitted with Eqs. 1 and 2 of the main text to take the segregation trailing edge into account. All other QW interfaces are well fitted by using Eq. 1 of the main text (i.e. constant r_b). The average values for each element and each interface reported in Tabs. S1 and S2 are discussed in the main text. $4\tau_i$ values are calculated with the approximation in Eqs. 3 and 4 of the main text, for comparison with other APT studies on this type of heterostructures (see Experimental Section of the main text).

Table S1: Fitted parameters and errors for two specimen (1 & 2) with focus on the top QW interface and for two specimen (1 & 2) with focus on the bottom QW interface. For the average values of the fitted parameters (rows ⟨Ge⟩ and ⟨Si⟩), the first error is the standard deviation based on the two specimen values. The second error is the propagated error of the average. $4\tau_i$ values are calculated with the approximation in Eqs. 3 and 4 of the main text.

	Name	r_t (nm)	$4\tau_{\mathrm{t}}$ (nm)	r_b (nm)	$4\tau_{\mathrm{b}}$ (nm)	r_{TE} (nm)	$4\tau_{\mathrm{TE}}$ (nm)	C_0 (%)
**Top interface:**								
	Ge (1)	1.13±0.05	2.0 ±0.09	0.7±0.5	1.3±0.9	4.5±0.3	8.0±0.5	0.34±0.1
	Ge (2)	0.76±0.03	1.35±0.05	1.5±0.4	2.7±0.7	5.6±0.2	9.9±0.4	0.39±0.1
	Si (1)	0.5±0.1	0.9±0.2	1.6±0.2	2.8±0.4			0.1±0.1
	Si (2)	0.8±0.1	1.4±0.2	2.2±0.3	3.9±0.5			0.1±0.1
	⟨Ge⟩	0.95±0.3±0.04	1.68±0.5±0.06	1.1±0.6±0.4	2.0±1.0±0.9	5.1±0.8±0.3	9.0±1.4±0.4	0.37±0.1
	⟨Si⟩	0.7±0.2±0.1	1.15±0.4±0.1	1.9±0.4±0.3	3.4±0.8±0.4			0.1±0.1
**Bottom interface:**								
	Ge (1)	2.15±0.08	3.8± 0.1	0.5±0.2	0.9±0.4	3.4±0.2	6.0±0.4	0.39±0.1
	Ge (2)	2.07±0.09	3.7±0.2	0.5±0.2	0.9±0.4	3.8±0.3	2.1±0.2	0.38±0.1
	Si (1)	1.0±0.2	1.8±0.4	0.6±0.1	1.1±0.2			0.1±0.1
	Si (2)	1.2±0.2	2.1±0.4	0.8±0.2	1.4±0.4			0.1±0.1
	⟨Ge⟩	2.11±0.06±0.09	3.7±0.1±0.1	0.5±0.0±0.2	0.9±0.0±0.4	3.6±0.3±0.3	6.4±0.5±0.5	0.39±0.1
	⟨Si⟩	1.1±0.1±0.1	2.0±0.3±0.4	0.7±0.1±0.2	1.2±0.3±0.3			0.1±0.1

Table S2: Fitted interface widths of the as-grown APT specimen. Fitted parameters and errors for two specimen (1 & 2) with focus on the top QW interface and for two specimen (1 & 2) with focus on the bottom QW interface. For the average values of the fitted parameters (rows ⟨Ge⟩ and ⟨Si⟩), the first error is the standard deviation based on the two specimen values. The second error is the propagated error of the average. $4\tau_i$ values are calculated with the approximation in Eqs. 3 and 4 of the main text. The quality of the Si isotope data of specimen 2 for the top interface did not allow to fit the Si concentration.

	Name	r'_t (nm)	$4\tau'_t$ (nm)	r'_b (nm)	$4\tau'_b$ (nm)	r'_{TE} (nm)	$4\tau'_{TE}$ (nm)	C_0 (%)
**Top interface:**								
	Ge (1)	0.62±0.02	1.1±0.04	0.8±0.3	1.4±0.5	3.8±0.1	6.7±0.2	0.15±0.1
	Ge (2)	0.68±0.04	1.21±0.07	1.5±0.4	2.7±0.7	3.4±0.2	6.0±0.4	0.16±0.1
	Si (1)	0.31±0.09	0.6±0.2	1.7±0.2	3.0±0.4			0.1±0.1
	⟨Ge⟩	0.65±0.04±0.03	1.15±0.08±0.06	1.2±0.5±0.4	2.0±0.9±0.6	3.6±0.3±0.2	6.4±0.5±0.3	0.16±0.1
	⟨Si⟩	0.31±0.09	0.6±0.2	1.7±0.2	3.0±0.4			0.1±0.1
**Bottom interface:**								
	Ge (1)	1.77±0.08	3.1±0.1	0.3±0.3	0.5±0.5	3.3±0.4	5.9±0.7	0.22±0.1
	Ge (2)	1.73±0.07	3.1±0.1	0.7±0.2	1.2±0.4	3.3±0.3	5.9±0.5	0.23±0.1
	Si (1)	0.6±0.2	1.1±0.4	1.3±0.4	2.3±0.7			0.1±0.1
	Si (2)	1.0±0.2	1.8±0.4	0.9±0.2	1.6±0.4			0.1±0.1
	⟨Ge⟩	1.75±0.03±0.08	3.1±0.05±0.2	0.5±0.3±0.3	0.9±0.5±0.5	3.3±0.0±0.4	5.9±0.0±0.6	0.23±0.1
	⟨Si⟩	0.8±0.3±0.2	1.4±0.5±0.4	1.1±0.3±0.3	1.9±0.5±0.6			0.1±0.1

References

(1) Kasper, E.; Heim, S. *Appl. Surf. Sci.* **2004**, *224*, 3.

(2) Boykin, T. B.; Klimeck, G.; Eriksson, M. A.; Friesen, M.; Coppersmith, S. N.; von Allmen, P.; Oyafuso, F.; Lee, S. *Appl. Phys. Lett.* **2004**, *84*, 115.

(3) Hollmann, A.; Struck, T.; Langrock, V.; Schmidbauer, A.; Schauer, F.; Leonhardt, T.; Sawano, K.; Riemann, H.; Abrosimov, N. V.; Bougeard, D.; Schreiber, L. R. *Phys. Rev. Appl.* **2020**, *13*, 034068.

(4) Wuetz, B. P. et al. *Nat. Commun.* **2022**, *13*, 7730.

(5) Losert, M. P.; Eriksson, M. A.; Joynt, R.; Rahman, R.; Scappucci, G.; Coppersmith, S. N.; Friesen, M. *Phys. Rev. B* **2023**, *108*, 125405.
